# Design Factors That Influence the Performance of Flight Intercept Traps for the Capture of Longhorned Beetles (Coleoptera: Cerambycidae) from the Subfamilies Lamiinae and Cerambycinae

**DOI:** 10.1371/journal.pone.0093203

**Published:** 2014-03-26

**Authors:** Jeremy D. Allison, Basu D. Bhandari, Jessica L. McKenney, Jocelyn G. Millar

**Affiliations:** 1 Department of Entomology, Louisiana State University Agricultural Center, LSU Campus, Baton Rouge, Louisiana, United States of America; 2 Department of Entomology, University of California Riverside, Riverside, California, United States of America; University of California, Berkeley, United States of America

## Abstract

In North America, cerambycid beetles can have significant ecological and economic effects on forest ecosystems, and the rate of introduction and/or detection of exotic species is increasing. Detection and survey programs rely on semiochemical-baited intercept traps which are often ineffective for large woodborers like cerambycid beetles. This study examined the effects of flight intercept trap design on the capture of cerambycid beetles in the subfamilies Lamiinae and Cerambycinae. These subfamilies are the two largest in the Cerambycidae and they include many of the most damaging cerambycid pests and species on regulatory watch lists in North America. This study demonstrates that intercept trap design, treatment of trap surfaces with a lubricant, and the type of collection cup all influence the capture of beetles from the subfamilies Lamiinae and Cerambycinae. It also demonstrates that the addition of a large lubricant-treated collar to the bottom funnel of a multiple-funnel trap significantly increases the capture of some Lamiinae. The best trap design for both subfamilies was a lubricant treated multiple-funnel [MF] trap equipped with a wet cup and lubricant treated large collar on the bottom funnel. This design captured between 4 and 14 times more Lamiinae and Cerambycinae than commercially-available MF and panel traps.

## Introduction

International movement of people and goods, and the associated high-risk pathways for biological invasions (e.g., air cargo, container, and refrigerated shipping) continue to increase globally, resulting in increased introductions of insect species outside of their native ranges [1]. The majority of these species do not become established and go unnoticed [2]. However, those that do establish can cause substantial economic and ecological damage [3], [4]. These invasive species represent one of the most significant threats to the health of forests in North America [5].

The larvae of large wood-boring insects feed cryptically in the wood, and their development can take months to years, facilitating their introduction in wood products, wooden packing materials, and nursery stock. Among invasive insect species, large woodborers (cerambycid and buprestid beetles, siricid wasps) represent one of the most serious threats to forest health globally [6]–[10] and it appears that their rate of detection, and likely their rate of introduction into new geographic areas, is increasing [1]. Cost estimates of the potential damage that might be caused by non-native forest insects, generated using a modeling approach suggest that among three major feeding guilds [wood- and phloem-borers, sap feeders, and foliage feeders], wood- and phloem boring insects have the highest associated costs [11]. In North America alone, recent introductions of the brown spruce borer *Tetropium fuscum* (Fabricius), the Asian longhorned beetle *Anoplophora glabripennis* (Motschulsky), the eucalyptus longhorned borers *Phoracantha semipunctata* (Fabricius) and *P. recurva* Newman, the emerald ash borer *Agrilus planipennis* Fairmaire, and the woodwasp *Sirex noctilio* F. are estimated to have resulted in hundreds of millions to billions of dollars in direct losses and costs of control and containment measures [5], [9], [12], [13].

There are three distinct population processes in biological invasions: arrival (transport of individuals to areas outside their native range), establishment (growth of populations to levels high enough that extinction is unlikely), and spread (expansion of an invading species' range) [14]. Invasive species management strategies integrate regulatory and preventive measures to limit the arrival of exotics, and eradication and containment tactics to limit establishment and spread of exotics post-entry [14]. The success of containment and eradication efforts is predicated on the development of tools for the survey and detection of low-density populations of the target species (e.g., effective attractants and traps have been crucial to the success of gypsy moth containment efforts [15], [16]). Effective survey and detection tools are essential because: 1) as populations of the invasive species increase, the probability of containment and eradication decreases; 2) successful containment and eradication requires that the distribution of the target species be accurately defined, on an ongoing basis; and 3) evaluation of the success of management efforts is not possible without good monitoring tools [14], [17]–[19].

Our ability to develop operational detection and survey programs for invasive cerambycid beetles is hindered by the lack of effective survey and detection tools. Existing surveillance methods for large woodborers rely primarily on visual inspection during aerial, drive-through, and ground surveys, or flight intercept traps baited with host volatiles. Visual surveys have limited power to detect low-density populations [20], and are expensive, laborious, and time-consuming [14]. Although monitoring traps work well for some bark and ambrosia beetles [21], they are not yet very effective for large woodborers. Despite the prominent status of both native and exotic cerambycids as pests of forest and urban trees, plantations, orchards, and lumber and wooden structures [22]–[24], until recently little was known about their chemical ecology. As recently as 10 years ago, pheromones had been reported for only nine out of the ∼35,000 described cerambycid species [24]. Volatile pheromones and related attractants are now known for >100 species [25], [26], and this number is increasing rapidly. Although some progress has been made in trap designs for cerambycid beetles [27]–[33], advances in this field have not paralleled the advances made in the identification of semiochemical attractants. There is little point in developing attractants if there are no effective traps in which to deploy them.

The commercial multiple-funnel [hereafter MF] traps that are widely used for bark and wood-boring insects do not work well for cerambycids [27]–[29], and the most effective trap can vary among species [32]. Alternative designs have outperformed MF traps, but few are available commercially and none are as easy to deploy as MF traps. Empirical work suggests that factors contributing to the poor performance of MF traps include lack of a prominent vertical silhouette, and poor collection and retention efficiencies, with attracted beetles often not falling into or escaping from traps [27]–[30].

Of the eight cerambycid subfamilies, the Cerambycinae and Lamiinae contain the most species [34], [35], and many of the most damaging cerambycid pests (e.g., Lamiinae: *Anoplophora* spp. Hope and *Monochamus* spp. Dejean; Cerambycinae: *Xylotrechus* spp. Chevrolat and *Phoracantha* spp. Newman) and many of the cerambycid species on the US Department of Agriculture's Animal and Plant Health Inspection Service (APHIS) and the Canadian Food Inspection Agency (CFIA) pest watch lists belong to these subfamilies. The goal of this study was to improve the performance of semiochemical-baited intercept traps for the detection and monitoring of cerambycid beetles from these two subfamilies. Specific objectives were: 1) to determine the effects of intercept trap design, the ability to land on the trap surface, and the type of collection receptacle on trap captures; 2) to determine whether intercept trap effectiveness is reduced by attracted beetles falling outside the collection cup; and 3) to integrate the results of objectives 1 and 2 to develop an improved trap design, and to compare this design to the commercially-available MF and flight intercept panel (hereafter panel) traps.

## Materials and Methods

### Field Experiments

A series of eight field trapping experiments was conducted to examine the effect of intercept trap design on captures of lamiine (experiments 1–4, conducted concurrently 8-July to 12-August, 2010) and cerambycine beetles (experiments 5–8, conducted concurrently 3-June to 7-July, 2011). Two subsequent experiments were conducted to compare the improved design for each subfamily to the commercially available MF and panel traps (both purchased from ConTech Enterprises Inc., Victoria, BC, Canada, hereafter Contech) (all MF traps used in all experiments were 8-unit). The subfamily Lamiinae was targeted by baiting all of the traps with semiochemicals (α-pinene, ipsdienol, and ipsenol) known to be attractive to *Monochamus* spp. [24], [36]–[38]. Experiments 5–8 used the same experimental designs [i.e., experiments 1 and 5, 2 and 6, 3 and 7, and 4 and 8 were identical] but targeted the subfamily Cerambycinae by baiting all of the traps with racemic 3-hydroxy-2-hexanone and (2R*, 3R*)-hexanediol, two common pheromones for species from this subfamily [26], [39]. Experiment 9, targeting the subfamily Lamiinae, was conducted 7-September to 20-October, 2011, and experiment 10 was conducted 6-September to 20-October, 2011, targeting Cerambycinae.

**Figure 1:**
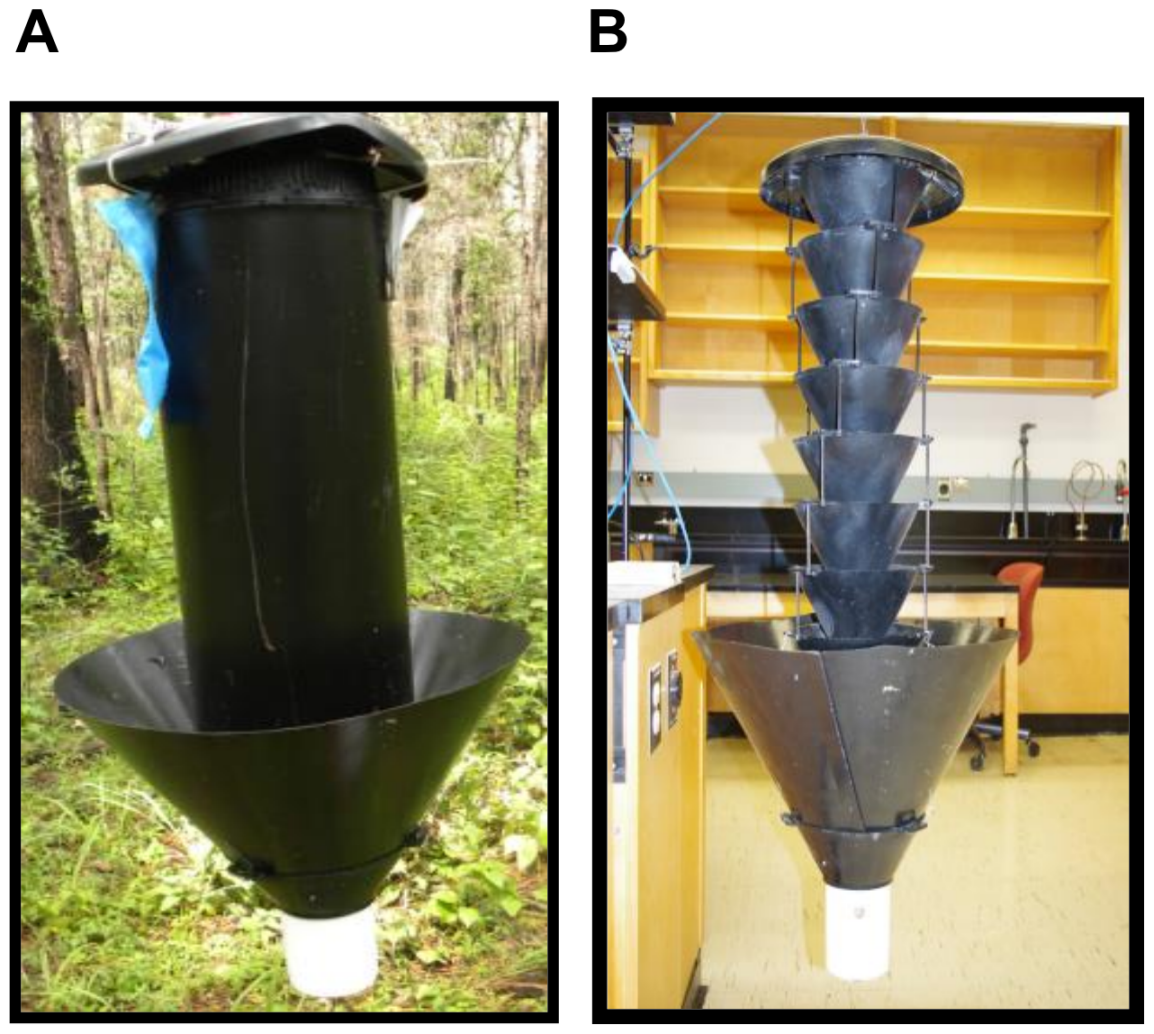
Images of the stovepipe trap used in Experiments 1 and 5 (Panel 1A) and the multiple-funnel trap with a collar added to the bottom funnel used in Experiments 4, 8, 9 and 10 (Panel 1B).

All of the field experiments used flight intercept traps deployed in a linear array of ten replicate blocks of three (Experiments 1, 3, 4, 5, 7, 8, 9, and 10) or six (Experiments 2 and 6) traps per block. Experiments 1 and 5 tested whether differences among the shape of different flight intercept traps influenced trap performance by comparing captures in: 1) commercial MF traps; 2) commercial panel traps; and 3) a modified MF trap with a custom made stovepipe replacing the concentric funnels. The stovepipe trap was fabricated using the top, and bottom funnel and collecting cup of a MF trap with a black stovepipe (diameter 20 cm, length 61 cm) inserted between the top and bottom funnel with the collecting cup attached. The bottom funnel was attached to a collar made from aluminum, painted black on both sides, that projected 25 cm at the same angle as the bottom funnel with the collection cup attached (i.e., the top of the collar was 10 cm above the bottom of the stovepipe) (maximum diameter of the collar was 48 cm) and hung from the bottom of the stovepipe so that there was an approximately 5 cm gap from the bottom of the stovepipe and the adjacent sides of the funnel (Fig. 1A). The three traps differed in the prominence of the silhouette presented [panel (80 cm length, 30 cm diameter) > stovepipe (61 cm length, 20 cm diameter) > MF (70 cm length, 20 cm in diameter funnel top and 11 cm in diameter at funnel bottom)], and all had a wet collection cup containing 150–200 ml of propylene glycol (ethanol free).

Experiments 2 and 6 tested whether treating trap surfaces with a lubricant influenced the number of cerambycids captured. Captures were compared in: 1) untreated MF traps; 2) MF traps treated with WorldKlass Dry Teflon Lubricant (hereafter Teflon) (part no. 00735, The WorldKlass Co., Nazareth, PA, USA); 3) MF traps treated with Fluon PTFE (hereafter Fluon; Northern Products Inc., Woonsocket RI, USA); 4) untreated intercept panel traps; 5) panel traps treated with Teflon; and 6) panel traps treated with Fluon. Teflon and Fluon were applied neat in the laboratory prior to trap deployment, allowed to air-dry and all trap surfaces were treated for complete coverage of collection surfaces. All of the MF and panel traps had wet collection cups containing 150–200 ml of propylene glycol.

Experiments 3 and 7 tested whether the retention of dry collection cups was improved by treating their interiors with Teflon. The treatments included: 1) MF trap with a wet collection cup containing 150–200 ml propylene glycol; 2) MF trap with a dry collection cup with an insecticide strip (3 cm × 3 cm × 0.15 cm HotShotNo-PestStrip, 18.6% dichlorvos, 20.4% inert ingredients); and 3) MF trap with a dry collection cup coated with Teflon (the interior of the dry collection cups were treated to complete coverage) and with an insecticide strip.

Experiments 4 and 8 tested the hypothesis that a subset of beetles striking the surface of the intercept trap fall outside the collection receptacle. The treatments were: 1) MF trap; 2) MF trap with an untreated collar; and 3) MF trap with a Fluon treated collar. The collars were identical to those used in Experiments 1 and 5 described above but were attached to a standard 8-unit MF trap (Fig. 1B). All traps had wet collection cups containing 150–200 ml propylene glycol.

**Figure 2:**
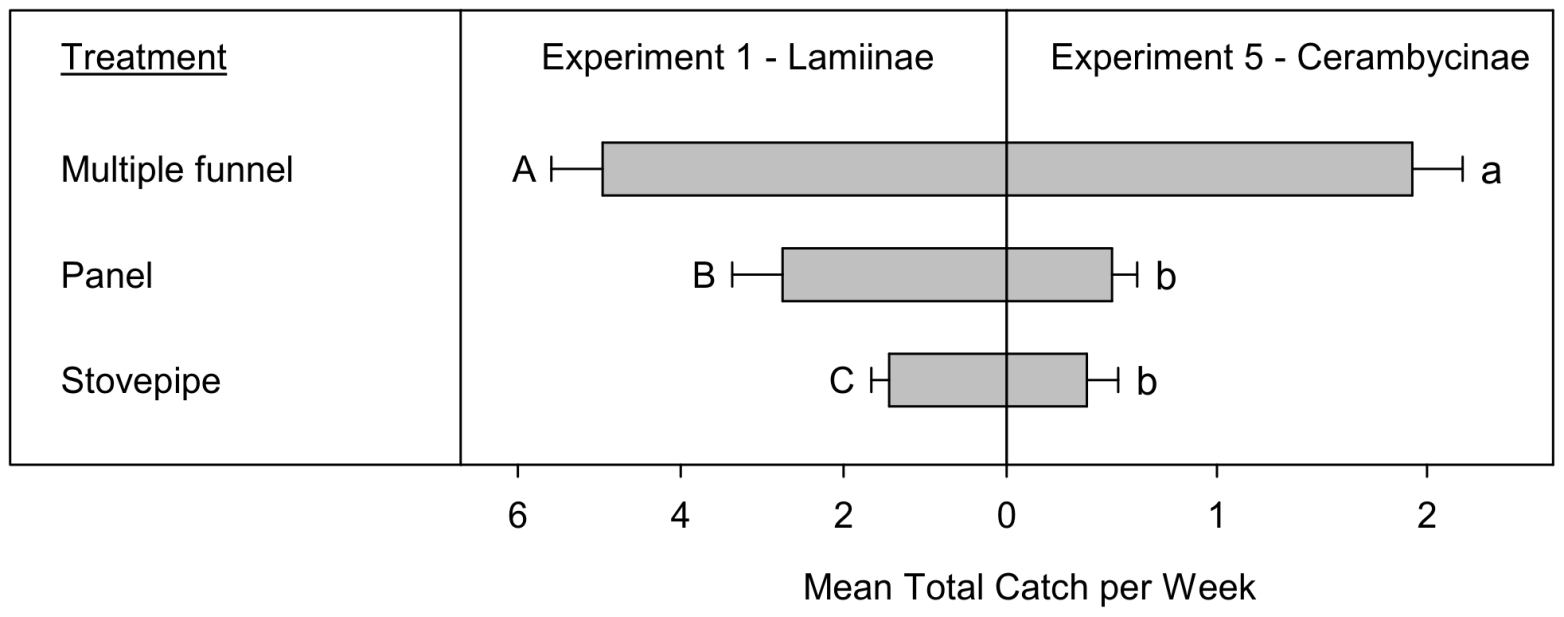
Mean total catches (+SE; N  =  10) per week of Lamiinae (Experiment 1) and Cerambycinae (Experiment 5) (species > 1 individual per trap captured). Means (+SE) followed by the same letter (uppercase: Lamiinae, lowercase: Cerambycinae) are not significantly different at *P* = 0.05. All traps in Experiment 1 were baited with 95% (−)-α-pinene and racemic ipsdienol and ipsenol. All traps in Experiment 5 were baited with racemic 3-hydroxy-2-hexanone and (2R*,3R*)-hexanediol.

**Figure 3:**
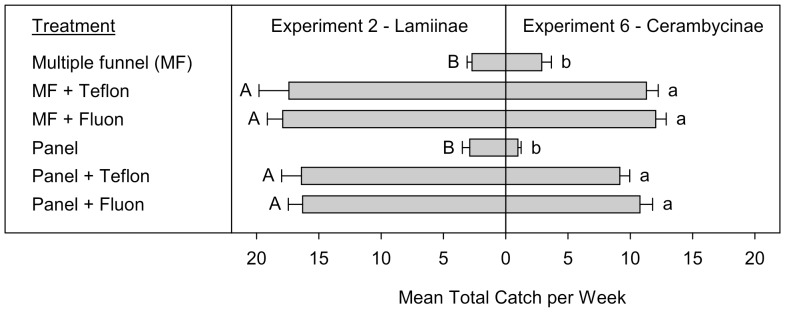
Mean total catches (+SE; N  =  10) per week of Lamiinae (Experiment 2) and Cerambycinae (Experiment 6) (species > 1 individual per trap captured). Means (+SE) followed by the same letter (uppercase: Lamiinae, lowercase: Cerambycinae) are not significantly different at *P* = 0.05. All traps in Experiment 2 were baited with 95% (−)-α-pinene and racemic ipsdienol and ipsenol. All traps in Experiment 6 were baited with racemic 3-hydroxy-2-hexanone and (2R*,3R*)-hexanediol.

**Figure 4:**
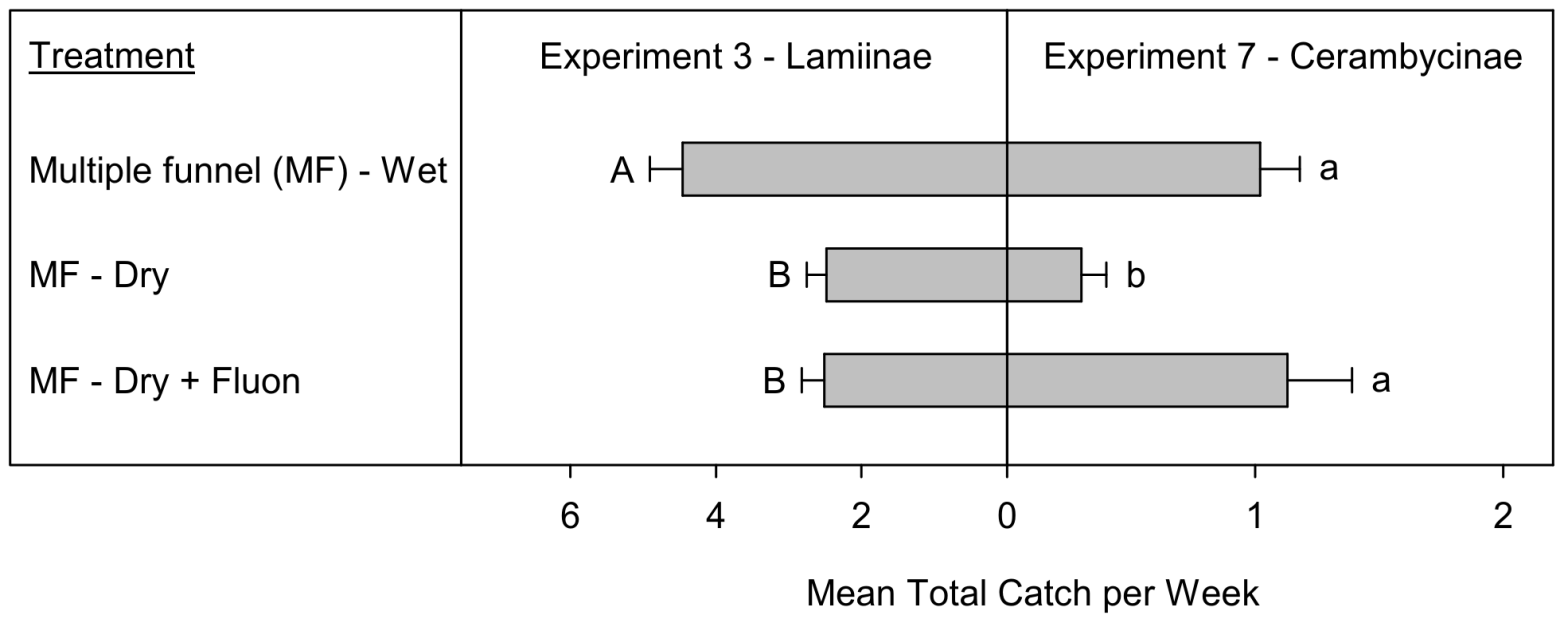
Mean total catches (+SE; N  =  10) per week of Lamiinae (Experiment 3) and Cerambycinae (Experiment 7) (species > 1 individual per trap captured). Means (+SE) followed by the same letter (uppercase: Lamiinae, lowercase: Cerambycinae) are not significantly different at *P* = 0.05. All traps in Experiment 3 were baited with 95% (−)-α-pinene and racemic ipsdienol and ipsenol. All traps in Experiment 7 were baited with racemic 3-hydroxy-2-hexanone and (2R*,3R*)-hexanediol.

**Figure 5:**
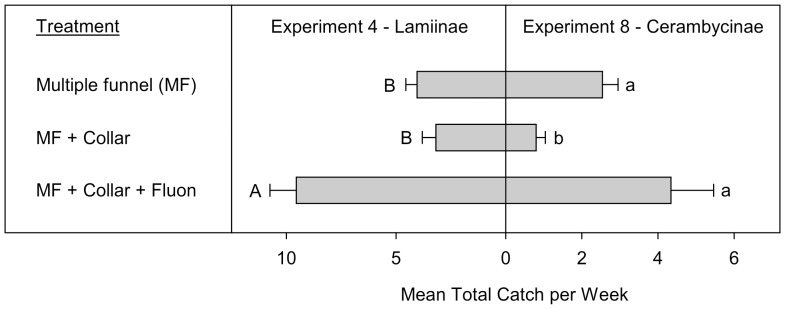
Mean total catches (+SE; N  =  10) per week of Lamiinae (Experiment 4) and Cerambycinae (Experiment 8) (species > 1 individual per trap captured). Means (+SE) followed by the same letter (uppercase: Lamiinae, lowercase: Cerambycinae) are not significantly different at *P* = 0.05. All traps in Experiment 4 were baited with 95% (−)-α-pinene and racemic ipsdienol and ipsenol. All traps in Experiment 8 were baited with racemic 3-hydroxy-2-hexanone and (2R*,3R*)-hexanediol.

Experiments 9 and 10 integrated the results of experiments 1-4 and 5–8 and compared the improved intercept design for the Lamiinae and Cerambycinae, respectively, to the commercially available MF and panel trap designs. The improved trap design for both subfamilies was identical (Fig. 2–5), i.e., MF trap treated with Teflon, with a wet collection cup and a Teflon treated collar. Experiments 9 and 10 had the following treatments: 1) standard MF trap; 2) standard intercept panel trap; and 3) MF trap treated with Teflon with a wet collection cup and Teflon treated collar.

All ten field experiments were conducted in the Kisatchie National Forest, Catahoula Ranger District, Louisiana, USA in stands of predominately *Pinus taeda* L. and mixed hardwoods that had experienced a prescribed burn preceding trap deployment in 2010 (the same stands were used in 2010 and 2011). We thank Anthony Page (Kisatchie National Forest, Catahoula Ranger District) for access to field sites. Traps were suspended individually from rope strung between two trees such that each trap was > 2 m from any tree and the collection cup of each trap was 0.5–1.5 m above the ground. All traps were at least 25 m apart within and between blocks. Species of Lamiinae and Cerambycinae were identified with standard keys [34], [35].

### Semiochemicals

Experiments 1–4 and 9 were baited with ultra-high release pouches containing α-pinene (172 ml; chemical purity ≥ 95%, enantiomeric purity 95% (−); release rate ≅ 2 g/d at 20°C) (Contech) as a representative host plant volatile [24] and bubble cap lures containing racemic ipsdienol and ipsenol with a chemical purity > 98% and release rates ≅ 0.1–0.2 mg/d at 25°C (Contech). Experiments 5–8 and 10 used lures loaded with racemic 3-hydroxy-2-hexanone prepared from 3-hydroxy-1-hexyne as described in [40] and (2R*,3R*)-2,3-hexanediol prepared by OsO_4_-catalyzed oxidation of (*E*)-2-hexene (GFS Chemicals, Powell, OH, USA), as described in [41]. In experiments 5–8, the 3-hydroxy-2-hexanone and (2R*, 3R*)-2,3-hexanediol lures were replaced on 20-June. These two compounds were diluted to concentrations of 50 mg/ml with ethanol and released individually from polyethylene sachets (Fisherbrand zipperseal sample bags, 51 micron wall thickness, 5 cm × 7 cm, Fisher Scientific, Pittsburgh, PA, USA) loaded with 1 ml of the respective solution. In experiment 9, the ipsenol and ipsdienol bubblecaps and α-pinene lures were all replaced on 23-September. In experiment 10 the 3-hydroxy-2-hexanone and (2R*,3R*)-2,3-hexanediol were replaced on 20-September.

### Statistical Analyses

Because the experimental designs were similar for all ten experiments [ten randomized complete blocks with multiple collection dates], the data were analyzed similarly. Total catches per trap of all Lamiinae and Cerambycinae were analyzed using a blocked multiresponse permutation procedure (MRBP) [42]. The catches from each collection period were summed by treatment for each subfamily. All analyses were conducted with PC-ORD 6.0 (MjM Software Design, Gleneden Beach, OR, USA) by using Euclidean distances to construct the distance matrix with blocks aligned before analysis [42]. The multiplicity effect was controlled using step-up FDR [43], [44].

## Results

In total 7,579 Lamiinae and 3,937 Cerambycinae were captured and included in statistical analyses in experiments 1–10 (see Table 1). Of the nine species of Lamiinae captured in experiments 1–4 and 9, *Monochamus titillator* (F.), *Monochamus carolinensis* Olivier, and *Acanthocinus obsoletus* (Olivier) were captured in high enough numbers (> 1 per trap) for analysis in all five experiments; *Acanthocinus nodosus* (Fabricius) was captured in high enough numbers for analysis in experiment 9 (see Table 2). The species *M. titillator*, *M. carolinensis*, *A. obsoletus*, and *A. nodosus* represented 64, 14, 20, and 1% of the total Lamiinae captured in experiments 1–4 and 9, respectively. In experiments 5–8 and 10, 15 species of Cerambycinae were captured, with *Neoclytus acuminatus* (Fabricius) and *Neoclytus mucronatus* (Fabricius) captured in high enough numbers (> 1 per trap) for analysis in all five experiments. *Neoclytus scutellaris* (Olivier) was captured in high enough numbers for analysis in experiments 5, 6, 8, and 10; *Xylotrechus colonus* (Fabricius) was captured in high enough numbers for analysis in experiments 6 and 10; *Elaphidion mucronatum* (Say) was captured in high enough numbers for analysis in experiments 6 and 8, and *Eburia quadrigeminata* (Say) was included in the analysis for experiment 6 (see Table 3). The species *N. acuminatus*, *N. mucronatus*, *N. scutellaris*, *X. colonus*, *E. mucronatum* and *E. quadrigeminata* represented 44, 23, 14, 4, 11, and 3% of the total Cerambycinae captured in experiments 5–8 and 10.

**Table 1:** Total number of individuals for all species of Lamiinae (Experiments 1–4 and 9)^1^ and Cerambycinae (Experiments 5–8 and 10)^2^ with > 1 individual captured per trap.

Experiment	Species	Total Captured
1	*Monochamus titillator*	340
	*Monochamus carolinensis*	39
	*Acanthocinus obsoletus*	42
2	*M. titillator*	2427
	*M. carolinensis*	550
	*A. obsoletus*	691
3	*M. titillator*	515
	*M. carolinensis*	52
	*A. obsoletus*	35
4	*M. titillator*	628
	*M. carolinensis*	91
	*A. obsoletus*	90
5	*Neoclytus acuminatus*	47
	*Neoclytus scutellaris*	37
	*Neoclytus mucronatus*	40
6	*N. acuminatus*	873
	*N. scutellaris*	291
	*N. mucronatus*	556
	*Eburia quadrigeminata*	74
	*Elaphidion mucronatum*	354
	*Xylotrechus colonus*	70
7	*N. acuminatus*	51
	*N. mucronatus*	57
8	*N. acuminatus*	148
	*N. scutellaris*	77
	*N. mucronatus*	75
	*E. mucronatum*	39
9	*M. titillator*	999
	*M. carolinensis*	361
	*A. obsoletus*	686
	*Acanthocinus nodosus*	33
10	*N. acuminatus*	701
	*N. scutellaris*	156
	*N. mucronatus*	221
	*X. colonus*	70

1 Experiments 1–4 and 9: all traps were baited with 95% (−)-α-pinene and racemic ipsenol and ipsdienol.

2 Experiments 5–8 and 10: all traps were baited with racemic 3-hydroxy-2-hexanone and (2R*,3R*)-hexanediol.

**Table 2:** Mean captures (± SE) by treatments for all species of Lamiinae (Experiments 1-4 and 9).^1^

Experiment/Species	Mean Capture ± SE per Treatment					
1	Multiple funnel (MF)	Panel	Stovepipe			
*Monochamus titillator*	4.19 ± 0.51	2.16 ± 0.50	1.04 ± 0.16			
*Monochamus carolinensis*	0.36 ± 0.10	0.22 ± 0.08	0.26 ± 0.09			
*Acanthocinus obsoletus*	0.41 ± 0.12	0.37 ± 0.09	0.14 ± 0.07			
Total Lamiinae	4.96 ± 0.63	2.75 ± 0.62	1.44 ± 0.22			
**2**	**MF**	**MF + Teflon**	**MF + Fluon**	**Panel**	**Panel + Teflon**	**Panel + Fluon**
*M. titillator*	2.18 ± 0.34	11.40 ± 1.50	11.90 ± 1.07	2.30 ± 0.54	10.19 ± 1.19	10.72 ± 0.76
*M. carolinensis*	0.28 ± 0.08	2.90 ± 0.57	2.50 ± 0.42	0.24 ± 0.08	2.70 ± 0.34	2.38 ± 0.25
*A. obsoletus*	0.24 ± 0.05	3.10 ± 0.40	3.50 ± 0.38	0.36 ± 0.08	3.50 ± 0.53	3.20 ± 0.41
Total Lamiinae	2.70 ± 0.39	17.40 ± 2.40	17.90 ± 1.24	2.88 ± 0.60	16.40 ± 1.59	16.30 ± 1.15
**3**	**MF - Wet**	**MF - Dry**	**MF - Dry + Fluon**			
*M. titillator*	3.36 ± 0.42	2.22 ± 0.24	2.10 ± 0.30			
*M. carolinensis*	0.73 ± 0.11	0.12 ± 0.04	0.21 ± 0.08			
*A. obsoletus*	0.37 ± 0.09	0.14 ± 0.05	0.21 ± 0.06			
Total Lamiinae	4.46 ± 0.45	2.48 ± 0.27	2.51 ± 0.31			
**4**	**MF**	**MF + Collar**	**MF + Collar + Fluon**			
*M. titillator*	3.20 ± 0.44	2.60 ± 0.53	7.25 ± 1.04			
*M. carolinensis*	0.36 ± 0.12	0.31 ± 0.08	1.20 ± 0.19			
*A. obsoletus*	0.48 ± 0.12	0.31 ± 0.09	1.10 ± 0.12			
Total Lamiinae	4.04 ± 0.51	3.18 ± 0.62	9.55 ± 1.20			
**9**	**MF**	**Panel**	**Optimal Design**			
*M. titillator*	4.10 ± 0.83	5.28 ± 0.86	15.58 ± 2.02			
*M. carolinensis*	1.30 ± 0.11	1.70 ± 0.25	6.03 ± 0.79			
*A. obsoletus*	2.98 ± 0.52	2.55 ± 0.59	11.63 ± 1.59			
*Acanthocinus nodosus*	0.18 ± 0.07	0.15 ± 0.09	0.50 ± 0.08			
Total Lamiinae	8.58 ± 1.36	9.68 ± 1.67	33.73 ± 3.93			

1 All traps were baited with 95% (−)-α-pinene and racemic ipsenol and ipsdienol.

**Table 3:** Mean captures (± SE) by treatments for all species of Cerambycinae (Experiments 5–8 and 10).^1^

Experiment/Species	Mean Capture ± SE per Treatment					
5	Multiple funnel (MF)	Panel	Stovepipe			
*Neoclytus acuminatus*	0.68 ± 0.15	0.16 ± 0.05	0.16 ± 0.05			
*Neoclytus mucronatus*	0.68 ± 0.14	0.11 ± 0.05	0.11 ± 0.05			
*Neoclytus scutellaris*	0.57 ± 0.14	0.23 ± 0.06	0.23 ± 0.06			
Total Cerambycinae	1.93 ± 0.24	0.50 ± 0.12	0.50 ± 0.12			
**6**	**MF**	**MF + Teflon**	**MF + Fluon**	**Panel**	**Panel + Teflon**	**Panel + Fluon**
*N. acuminatus*	1.05 ± 0.43	4.82 ± 0.68	5.37 ± 0.81	0.39 ± 0.20	2.92 ± 0.47	3.94 ± 0.54
*N. mucronatus*	0.76 ± 0.26	2.94 ± 0.51	3.09 ± 0.72	0.12 ± 0.05	2.18 ± 0.35	2.85 ± 0.53
*N. scutellaris*	0.49 ± 0.16	1.38 ± 0.25	1.50 ± 0.38	0.06 ± 0.03	1.38 ± 0.23	1.43 ± 0.17
*Xylotrechus colonus*	0.06 ± 0.04	0.16 ± 0.07	0.34 ± 0.08	0.02 ± 0.02	0.56 ± 0.20	0.35 ± 0.11
*Elaphidion mucronatum*	0.40 ± 0.12	1.56 ± 0.35	1.31 ± 0.18	0.31 ± 0.08	1.79 ± 0.24	2.09 ± 0.53
*Eburia quadrigeminata*	0.13 ± 0.04	0.43 ± 0.13	0.43 ± 0.18	0.08 ± 0.03	0.33 ± 0.10	0.12 ± 0.07
Total Cerambycinae	2.89 ± 0.78	11.29 ± 0.95	12.04 ± 0.84	0.98 ± 0.26	9.16 ± 0.79	10.78 ± 1.01
**7**	**MF - Wet**	**MF - Dry**	**MF - Dry + Fluon**			
*N. acuminatus*	0.36 ± 0.11	0.23 ± 0.11	0.56 ± 0.18			
*N. mucronatus*	0.66 ± 0.10	0.07 ± 0.03	0.57 ± 0.22			
Total Cerambycinae	1.02 ± 0.16	0.30 ± 0.10	1.13 ± 0.26			
**8**	**MF**	**MF + Collar**	**MF + Collar + Fluon**			
*N. acuminatus*	0.79 ± 0.19	0.34 ± 0.20	2.21 ± 0.73			
*N. mucronatus*	0.68 ± 0.25	0.18 ± 0.08	0.84 ± 0.21			
*N. scutellaris*	0.75 ± 0.10	0.23 ± 0.05	0.77 ± 0.12			
*E. mucronatum*	0.32 ± 0.10	0.04 ± 0.03	0.52 ± 0.25			
Total Cerambycinae	2.54 ± 0.41	0.80 ± 0.24	4.34 ± 1.12			
**10**	**MF**	**Panel**	**Optimal Design**			
*N. acuminatus*	2.20 ± 0.35	0.90 ± 0.30	10.90 ± 1.87			
*N. mucronatus*	0.74 ± 0.21	0.20 ± 0.07	3.48 ± 0.46			
*N. scutellaris*	0.44 ± 0.18	0.14 ± 0.06	2.54 ± 0.55			
*X. colonus*	0.24 ± 0.07	0.06 ± 0.03	1.10 ± 0.23			
Total Cerambycinae	3.62 ± 0.59	1.30 ± 0.28	18.04 ± 2.16			

1 All traps were baited with racemic 3-hydroxy-2-hexanone and (2R*,3R*)-hexanediol.

Experiments 1 and 5 compared the performance of different intercept trap designs for the Lamiinae and Cerambycinae, respectively. In total, 421 Lamiinae (340 *M. titillator*, 39 *M. carolinensis*, and 42 *A. obsoletus*) and 124 Cerambycinae (47 *N. acuminatus*, 37 *N. scutellaris*, and 40 *N. mucronatus*) were included in the analyses in experiments 1 and 5, respectively (Table 1). There was a significant treatment effect on captures of both the total Lamiinae (Exp. 1: T  =  −6.1, *P*  =  0.00027) and Cerambycinae (Exp. 5: T  =  −6.9, *P*  =  0.00017). The overall pattern of intercept trap design effect on trap capture was similar for beetles from both subfamilies (Fig. 2). For both subfamilies, the MF trap captured significantly more individuals than the panel and stovepipe designs. The panel trap captured significantly more Lamiinae than the stovepipe trap.

Experiments 2 and 6 examined the effect of treating MF and panel traps with lubricants on the capture of Lamiinae and Cerambycinae, respectively. In total 3,668 Lamiinae (2,427 *M. titillator*, 550 *M. carolinensis*, and 691 *A. obsoletus*) and 2,218 Cerambycinae (873 *N. acuminatus*, 291 *N. scutellaris*, 556 *N. mucronatus*, 74 *E. quadrigeminata*, 354 *E. mucronatum*, and 70 *X. colonus*) were included in the analyses of experiments 2 and 6, respectively. There was a significant treatment effect for both the total Lamiinae (Exp. 2: T  =  −13.6, *P* < 0.0001) and Cerambycinae captured (Exp. 6: T  =  −13, *P* < 0.0001). The overall pattern of trap captures was identical for both subfamilies (Fig. 3), with treatment of MF and panel traps with Teflon or Fluon lubricant resulting in significant increases in captures, whereas there were no differences among treated MF and panel traps, or between untreated MF and panel traps.

Experiments 3 and 7 tested the effect of collection cup design on the capture of Lamiinae and Cerambycinae, respectively. In total, 602 Lamiinae (515 *M. titillator*, 52 *M. carolinensis*, and 35 *A. obsoletus*) and 108 Cerambycinae (51 *N. acuminatus* and 57 *N. mucronatus*) were included in the analyses of experiments 3 and 7, respectively. There was a significant treatment effect on both the total Lamiinae (Exp. 3: T  =  −4.4, *P* < 0.003) and Cerambycinae captured (Exp. 7: T  =  −2.7, *P* < 0.02). The overall pattern of treatment effects differed between the two subfamilies (Fig. 4). Although MF traps with a wet cup captured significantly more Lamiinae and Cerambycinae than MF traps with a dry cup, treatment of the interior of dry cups with Teflon significantly improved the performance of dry cups for retaining Cerambycinae but not Lamiinae.

Experiments 4 and 8 tested the effects of adding a large collar to the bottom of MF traps on the capture of Lamiinae and Cerambycinae, respectively. Totals of 809 Lamiinae (628 *M. titillator*, 91 *M. carolinensis*, and 90 *A. obsoletus*) and 339 Cerambycinae (148 *N. acuminatus*, 77 *N. scutellaris*, 75 *N. mucronatus*, and 39 *E. mucronatum*) were included in the analyses of experiments 4 and 8, respectively. There was a significant treatment effect on both the total Lamiinae (Exp. 4: T  =  −5.7, *P* < 0.0007) and Cerambycinae captured (Exp. 8: T  =  −5.6, *P* < 0.0006). For both subfamilies, MF traps with a Fluon treated collar captured significantly more individuals than MF traps with an untreated collar (Fig. 5). Whereas there was no difference in the number of Cerambycinae captured by MF traps and MF traps with a Fluon treated collar, MF traps with a Fluon treated collar captured significantly more Lamiinae than MF traps.

**Figure 6:**
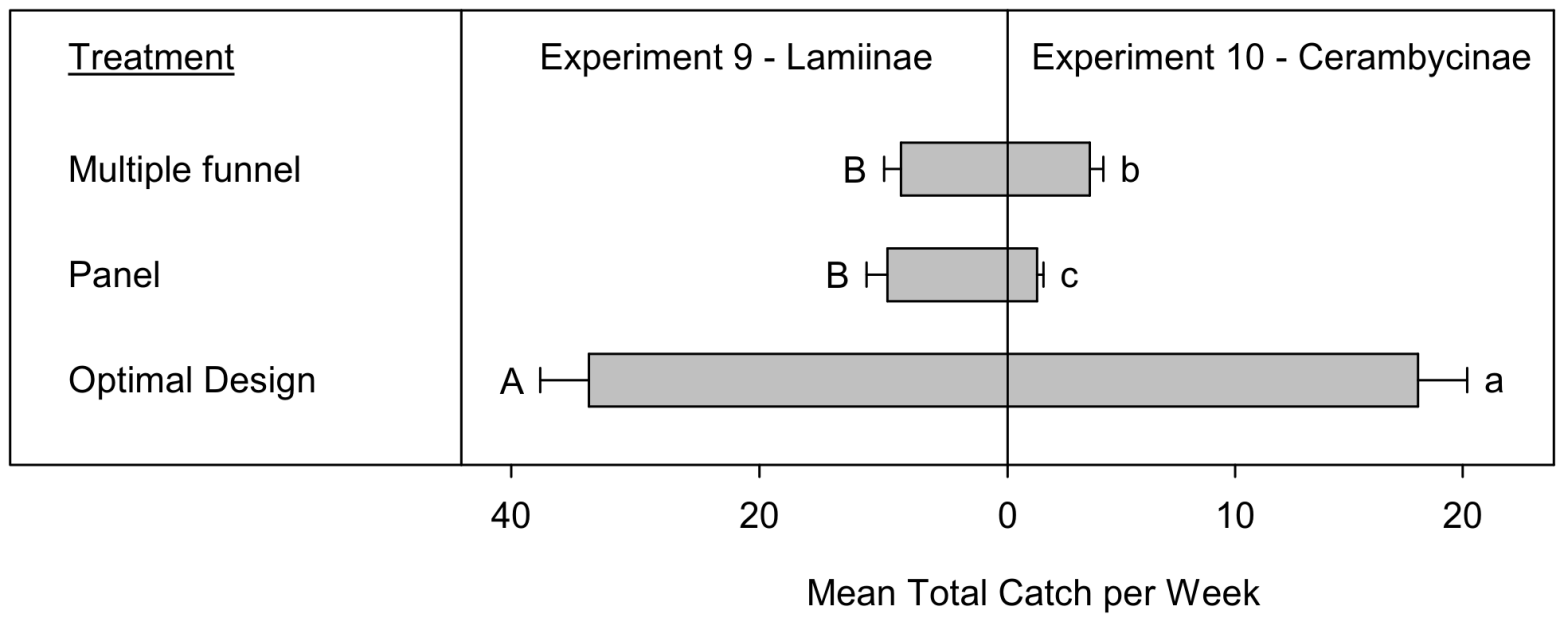
Mean total catches (+SE; N  =  10) per week of Lamiinae (Experiment 9) and Cerambycinae (Experiment 10) (species > 1 individual per trap captured). Means (+SE) followed by the same letter (uppercase: Lamiinae, lowercase: Cerambycinae) are not significantly different at *P* = 0.05. All traps in Experiment 9 were baited with 95% (−)-α-pinene and racemic ipsdienol and ipsenol. All traps in Experiment 10 were baited with racemic 3-hydroxy-2-hexanone and (2R*,3R*)-hexanediol.

Experiments 9 and 10 compared the improved intercept trap design based on the results of experiments 1–4 and 5–8, to the commercially available MF and panel traps, for the Lamiinae and Cerambycinae, respectively. For both subfamilies the best design was a Fluon treated MF trap with a wet cup and Fluon treated collar. In total 2,079 Lamiinae (999 *M. titillator*, 361 *M. carolinensis*, 686 *A. obsoletus*, and 33 *A. nodosus*) and 1,148 Cerambycinae (156 *N. scutellaris*, 221 *N. mucronatus*, 701 *N. acuminatus*, and 70 *X. colonus*) were included in the analyses of experiments 9 and 10. There was a significant treatment effect on both the total Lamiinae (Exp. 9: T  =  −7.5, *P* < 0.0001) and Cerambycinae captured (Exp. 10: T  =  −9.2, *P* < 0.0001). For both subfamilies the improved trap design captured significantly more individuals than either the MF or panel traps (Fig. 6), and untreated MF traps captured significantly more Cerambycinae than untreated panel traps.

## Discussion

Because of their ease of use and cost-effectiveness, semiochemical-baited flight intercept traps are used extensively in surveillance, mass trapping, and monitoring programs for cerambycid beetles, and in research trials [37], [45]–[47]. Numerous studies have identified trap design features that influence the attraction, capture, and retention of wood-boring and bark beetles in flight intercept traps [27]–[30], and the cumulative results from these studies suggested that the performance of commercially available flight intercept traps could be improved. This study demonstrated that straightforward modifications to commercial trap designs can substantially increase the capture and retention of lamiine and cerambycine beetles.

Several studies have suggested that MF trapping efficiency for cerambycids is low compared to other designs, likely due to differences in the vertical silhouette presented by the various designs [27]–[30], [48], [49]. Alternatively, differences among trap types may be due to variation in the ability of cerambycid beetles to gain purchase on the trap surface. Unfortunately, most of these studies had confounding effects among treatments in collection methods (e.g., sticky stovepipe vs. dry collection container) [48] or the size and type of collection container [27], [29], [49]. Consequently, the importance of silhouette relative to other design factors remained unclear. de Groot and Nott [28] demonstrated an effect of silhouette (presence/absence) on trapping effectiveness but did not include the MF trap design in their study. Morewood et al. [30] compared a panel trap design to MF traps modified to have the same collection container and observed that for most cerambycid species, the panel trap was more effective than the MF trap design. Unlike previous studies which reported no differences in the effectiveness of panel and MF traps [29], [49] or higher captures by panel (or panel-like) traps [27], [29], [30], this study found that captures of both lamiine and cerambycine beetles were significantly higher in MF than panel traps. For the subfamily Lamiinae, this study also found that MF and panel traps were superior to stovepipe traps, whereas for the Cerambycinae, only the MF trap was superior to the stovepipe design. The reason for the higher captures in the MF traps was unclear. There is some evidence that suggests that lure location influences both the odour plume of intercept traps [50] and their performance [51]. It is possible that differences in the odour plumes of the three intercept trap designs contributed to differences in cerambycid capture observed in this study. Alternatively, or in addition to silhouette and odour plume differences, the MF trap may capture and/or retain a higher portion of those individuals attracted to the trap.

Several studies have examined whether the ability to land on the trap surface influences cerambycid captures in flight intercept traps [29], [49], [52]–[54]. These studies compared captures in untreated flight intercept traps to traps treated with various surface lubricants and observed that for both panel [29], [49], [53] and MF traps [54], lubricant treated traps captured more cerambycids than untreated traps, but see [52]. The results of this study support the existing literature and demonstrate a significant positive effect of treating both MF and panel traps with either Fluon or Teflon lubricant to increase capture efficiency for lamiine and cerambycine species. Furthermore, the sprayable (and easier to apply) Teflon lubricant was comparable to the liquid fluoropolymer Fluon. The cumulative results from these studies suggest that many of the cerambycid beetles striking/landing on untreated flight intercept trap surfaces are not captured.

Escape of insects from the collection container also can decrease the effectiveness of flight intercept traps. Typically, flight intercept traps use either a dry collection cup containing a killing agent (e.g., an insecticide-impregnated strip) or a wet cup partially filled with a liquid (e.g., soapy water, salt solution, propylene glycol) to act as a killing and preservation agent. Several studies have demonstrated that flight intercept traps with wet collection cups capture more cerambycids than traps with dry collection cups [29]–[31], [33], a result also observed in this study. The effect of the cup type is likely due to individuals escaping from dry cups, in contrast to few or no escapes from wet cups before drowning. For example, Nakamura et al. [55] reported a 30% daily loss of *M. alternatus* Hope (subfamily Lamiinae) from intercept traps without a killing agent. Assuming that the difference between the mean captures of wet and dry cups in this study is due to escapes, 49% of the lamiines and 71% of the cerambycines escaped from dry cups. Nevertheless, although dry traps captured fewer individuals than wet traps, the former are easier to use operationally. Graham et al. [53] tested whether the performance of dry cups could be improved by coating their interiors with Fluon or the polysiloxane liquid Rain-X. They observed that *Megacyllene robiniae* [Forster] (subfamily Cerambycinae) was four times more likely to escape from untreated or Rain-X treated cups than Fluon treated cups. Overall, this study showed that traps with Fluon treated dry cups captured far more Cerambycinae than traps with untreated dry cups, and significantly, that Fluon treated dry cups were equivalent in performance to traps with wet cups for cerambycine beetles. Surprisingly, treating dry cups with Fluon had no effect on retention efficiency of lamiines.

Researchers working on the management and chemical ecology of cerambycid beetles have hypothesized that trapping efficiency is decreased by attracted individuals striking the trap but not dropping into the collectors. This hypothesis was tested by adding large diameter collars to the bottom funnel of MF traps [29], [30]. Whereas neither study observed an effect for lamiine species, *Xylotrechus longitarsus* Casey (subfamily Cerambycinae) and male *Arhopalus asperatus* (LeConte) (subfamily Aseminae) were trapped more effectively in traps fitted with collars [30]. Empirical work comparing the captures of intercept traps treated with lubricants to untreated traps suggests that the effect of the collar may have been diminished by beetles being able to walk on the collars and escape capture. Although they did not add a collar to flight intercept traps, in a laboratory bioassay Graham et al. [53] observed that whereas *M. robiniae* were able to walk up Rain-X or untreated bases of panel traps, they were unable to walk up the base of a panel trap treated with Fluon. The current study found that the addition of an untreated collar to MF traps had no effect on the total number of lamiines captured, and significantly reduced captures of cerambycines. In contrast, addition of a Fluon-treated collar significantly increased the capture of Lamiinae while having no effect on captures of cerambycines relative to untreated MF traps.

In summary, the results of this study demonstrate that the shape of different types of intercept trap, treatment of intercept trap surfaces with a lubricant, and the type of collection container can all influence the capture of beetles in the subfamilies Lamiinae and Cerambycinae. They also suggest that a subset of the lamiine beetles that are attracted to MF traps, strike the trap surface but fall outside the collection cup or that the addition of a large collar results in more beetles being attracted to multiple-funnel traps. Cumulatively, these results suggest that the best trap design for beetles from both subfamilies is a lubricant treated trap equipped with a wet collection cup and a large diameter, Fluon-treated collar on the bottom funnel and that the MF trap is at least as good or better than panel traps. This improved design captured 4 to 14 times more Lamiinae and Cerambycinae than commercially available MF and panel traps.
